# National and Individual‐Level Economic Burden of Local Corticosteroid Use and Concomitant Use of Multiple Local Corticosteroid Formulations—A Nationwide Cohort Study

**DOI:** 10.1111/cea.70351

**Published:** 2026-05-21

**Authors:** Kjell Erik Julius Håkansson, Jonas Bækdal, Inge Raadal Skov, Steven Arild Wuyts Andersen, Zarqa Ali, Anders Løkke, Rikke Ibsen, Ole Hilberg, Howraman Meteran, Claus Rikard Johnsen, Vibeke Backer, Charlotte Suppli Ulrik

**Affiliations:** ^1^ Department of Respiratory Medicine Copenhagen University Hospital—Hvidovre Hvidovre Denmark; ^2^ Department of Respiratory Medicine Copenhagen University Hospital—Bispebjerg Copenhagen Denmark; ^3^ Department of Medicine Lille Baelt Hospital Kolding Denmark; ^4^ Department of Otorhinolaryngology—Head & Neck Surgery and Audiology Copenhagen University Hospital—Rigshospitalet Copenhagen Denmark; ^5^ Institute of Clinical Medicine University of Copenhagen Copenhagen Denmark; ^6^ Department of Dermatology Copenhagen University Hospital—Bispebjerg Copenhagen Denmark; ^7^ Department of Medicine Lille Baelt Hospital Vejle Denmark; ^8^ Department of Regional Health Research University of Southern Denmark Odense Denmark; ^9^ i2Minds Aarhus Denmark; ^10^ Allergy Clinic Copenhagen University Hospital—Gentofte Gentofte Denmark

## Abstract

- Approximately one third of Danish adults use local corticosteroids, associated with billions in annual societal costs.
- Concomitant use of multiple local corticosteroid formulations is associated with increased financial burden.

To the Editor,

Local corticosteroid treatment, including inhaled, nasal and topical formulations, is the backbone of many highly prevalent diseases such as asthma, allergic rhinitis, chronic rhinosinusitis and atopic dermatitis [1, 2]. Local corticosteroids are typically well tolerated. However, prolonged high‐dose local corticosteroid exposure can contribute to adverse effects typically seen with systemic corticosteroid exposure [3, 4]. As some of the above‐mentioned diseases share inflammatory pathways (e.g., type 2 inflammation), patients may be exposed to multiple routes of administration [5]. Despite their ubiquity, the financial impact of individuals using one or more formulations of local corticosteroids has been sparsely investigated. In the present study, we aimed to quantify the healthcare and welfare burden on a societal level, as well as the loss of personal income for individuals using one or more formulations of local corticosteroids as compared to matched comparators.

Using the Danish national registries covering all residents of Denmark, we identified incident local corticosteroid users based on repeated pharmacy redemptions (> 1 annual) or relevant ICD‐10 diagnoses such as asthma or allergic rhinitis between 2002 and 2020. Excess costs compared to age‐, sex‐, residence‐ and marital status‐matched comparators were calculated using national registries on healthcare utilization (direct costs e.g., hospital admission, medications), welfare transfers (e.g., sick leave) and loss of income (differences in salaried wage). Costs were estimated using generalized linear regression models (GLM) with a gamma distribution and a log link [6]. Costs are reported as excess Euro (95% CI) and are adjusted for education level, comorbidity burden (Charlson comorbidity index using prescription patterns and diagnostic codes) and systemic corticosteroid‐treated comorbidity (e.g., cancers, IBD). Welfare transfers were estimated for individuals in the working age (18–64). For full national financial burden estimation, the difference unadjusted crude means were used. Full cohort definitions, cost subdivisions and methods are available in an online repository (http://doi.org/10.5281/zenodo.19651707).

In all, 1,935,349 adults who redeemed repeated prescriptions for local corticosteroids and 3,821,720 comparators were included. Multiple administration routes were common, with 21% redeeming two concomitant formulations, and 3% redeeming three. The median age was 50 [IQR 33], 54 [30] and 55 [29] years, and 54%, 58% and 61% were female, respectively.

Annual excess healthcare costs exhibited a clear gradient across number of formulations. For users of one, two and three formulations the costs were 755 (95% CI 752–757), €1667 (1658–1675) and €2661 (2629–2693), respectively (Figure 1, Panel A). Outpatient care was the largest expenditure for individuals redeeming one local corticosteroid (€218 (217–219)), whereas hospitalisations were the largest contributor for those redeeming multiple formulations (€431 (429–433) and €682 (673–691), respectively). Of note, the annual excess costs associated with corticosteroid exposure‐associated diagnoses were €25 (25–25), €54 (54–55) and €83 (82–84) for individuals using one, two and three formulations, respectively (Figure 1, Panel B).

**FIGURE 1:**
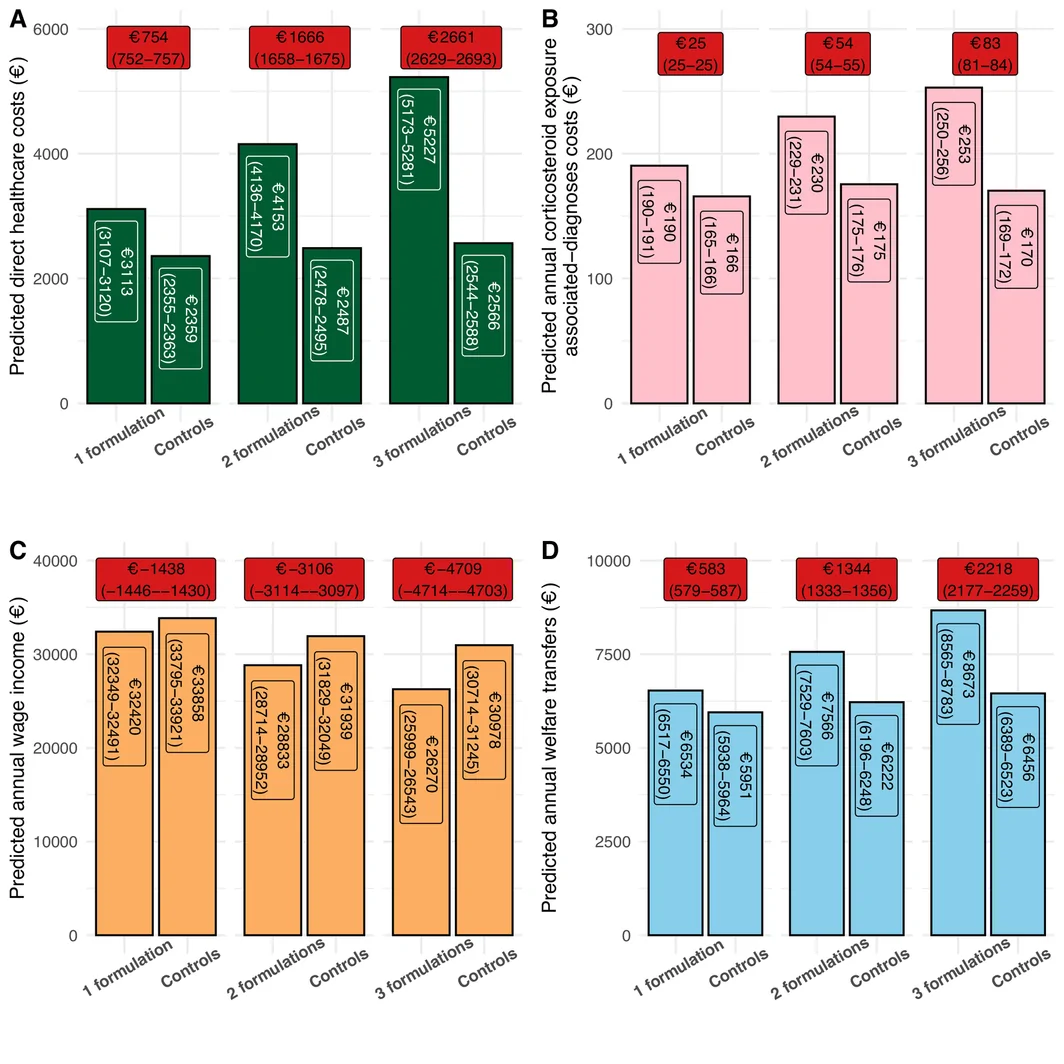
Predicted financial burden for 1,935,349 Danish adults redeeming one or more formulation of local corticosteroids and their matched comparators, stratified by (A) Direct healthcare costs, (B) Corticosteroid exposure associated‐diagnoses direct healthcare costs, (C) Wage income and (D) Welfare transfers. Differences between cases and comparators in red boxes.

Loss of income demonstrated similar patterns of increasing costs with increasing number of formulations used at €1438 (1430–1446), €3106 (3097–3114) and €4709 (4703–4714) for one, two and three formulations, respectively (Figure 1, Panel C). Excess welfare transfers for working‐age individuals redeeming one, two and three formulations were €583 (579–587), €1344 (1333–1356) and €2218 (2177–2259) (Figure 1, Panel D). Disability pensions were the largest contributor to excess welfare transfers at €307 (307–309), €830 (825–835) and €1530 (1509–1552).

From a societal perspective, the pooled annual excess direct healthcare costs associated with local corticosteroid use amounted to €2.27 billion, of which the annual costs associated with corticosteroid exposure‐associated diagnoses constituted €82.53 million. Additionally, a total annual income loss of €2.77 billion and €1.46 billion in excess welfare transfers were observed.

The present study of nearly 2 million users of local corticosteroids demonstrates that the diseases associated with local corticosteroid use—and especially concomitant multiple formulation use—impose a substantial financial burden. One in four individuals was treated with multiple formulations and an increase in financial burden was observed with increasing numbers of formulations used.

Individual‐level excess direct costs for patients treated with a single formulation were consistent with previous findings on mild asthma and allergic rhinitis [7, 8], two common indications for local corticosteroids. The marked increase in excess costs seen in users of multiple formulations likely reflects greater disease severity and cumulative healthcare utilisation due to multimorbidity. The shift from predominantly outpatient expenditure in users of a single local corticosteroid to hospitalisation‐driven costs among those with concomitant use of multiple corticosteroids further suggests that use patterns reflect increased disease complexity and burden. Furthermore, the diseases treated with local corticosteroids had a significant financial impact on treated individuals' attachment to the workforce and personal income, suggesting further room for improving disease control despite local corticosteroid use—especially considering the high prevalence of use in the general population.

Of note, the corticosteroid exposure‐associated excess costs were generally low per individual, but from a societal perspective still constitute a significant financial burden—albeit not at the same level as seen in, for example, severe asthma where prolonged systemic corticosteroid treatment is common [9]. While local corticosteroid formulations are generally cost‐effective, optimising background therapy and the emergence of corticosteroid‐sparing treatments such as biologics, sinus surgery or Janus kinase inhibitors may reduce disease‐related costs in patients with severe disease presentations.

Limitations of the present study include potential misclassification of milder disease presentations due to over‐the‐counter availability of some treatments, and the inability to distinguish singular disease entities. Future longitudinal studies should assess the cumulative cost impact of prolonged local corticosteroid exposure across multiple routes of administration.

## Author Contributions

Conception and design (K.E.J.H., J.B., I.R.S., S.A.W.A., A.L., O.H., H.M., C.R.J., V.B., C.S.U.); analysis (K.E.J.H., R.I., J.B.); interpretation (K.E.J.H., J.B., I.R.S., S.A.W.A., A.L., O.H., H.M., C.R.J., V.B., C.S.U.); first draft (K.E.J.H.); revisions, final approval and accountability (K.E.J.H., J.B., I.R.S., S.A.W.A., A.L., O.H., H.M., C.R.J., V.B., C.S.U.).

## Funding

This study was funded by an unrestricted research grant from AstraZeneca and SanofiGenzyme.

## Conflicts of Interest

K.E.J.H. has received personal fees from AstraZeneca, Chiesi, GSK, Sanofi and TEVA. A.L. has received personal fees from AstraZeneca, GSK, TEVA, Chiesi, Sanofi Genzyme, Boehringer‐Ingelheim, Orion Pharma, Novartis, ALK‐Abello, Mundipharma and Pfizer. J.B. reports personal honoraria from NovoNordisk. I.R.S., R.I. and C.R.J. has no conflicts to declare. O.H. has received personal fees AstraZeneca, GSK, TEVA, Chiesi, Sanofi Genzyme and Boehringer‐Ingelheim. V.B. GSK, Sanofi Genzyme, AstraZeneca, TEVA, Chiesi, Boehringer‐Ingelheim, Novartis, ALK‐Abello, Mundipharma Menarini, Birk and Pfizer. S.A. has consulted for Ambu A/S C.S.U. has received personal fees from AstraZeneca, GSK, Sanofi, Novo Nordisk, Chiesi, Boehringer Ingelheim, Mundipharma, Pfizer, Berlin Chemie Menarini, Hikma Pharmaceuticals, TEVA, Orion Pharma, Novartis, TFF Pharmaceuticals, IQVIA and Roche and Actelion outside the submitted work.

## Data Availability

The data that support the findings of this study are available from Statistics Denmark. Restrictions apply to the availability of these data, which were used under licence for this study. Data are available from http://www.dst.dk with the permission of Statistics Denmark.
